# Therapeutic Investigation of Palm Oil Mill Effluent-Derived Beta-Carotene in Streptozotocin-Induced Diabetic Retinopathy via the Regulation of Blood–Retina Barrier Functions

**DOI:** 10.3390/ph16050647

**Published:** 2023-04-26

**Authors:** Yamunna Paramaswaran, Aswinprakash Subramanian, Nallupillai Paramakrishnan, Muthusamy Ramesh, Arunachalam Muthuraman

**Affiliations:** 1 PG Research Scholar, Faculty of Pharmacy, AIMST University, Semeling, Bedong 08100, Kedah, Malaysia; 2 Anatomy Unit, Faculty of Medicine, AIMST University, Bedong 08100, Kedah, Malaysia; 3 Department of Pharmacognosy, JSS College of Pharmacy, Mysore, JSS Academy of Higher Education and Research, Mysore 570015, Karnataka, India; nparamakrishnan@jssuni.edu.in; 4 Department of Pharmaceutical Analysis, Omega College of Pharmacy, Hyderabad 501301, Telangana, India; 5 Unit of Pharmacology, Faculty of Pharmacy, AIMST University, Bedong 08100, Kedah, Malaysia

**Keywords:** catalase, dexamethasone, macular degeneration, optomotor response, oxidative stress, reduced glutathione

## Abstract

Diabetic retinopathy (DR) primarily progresses into retinal degeneration caused by microvascular dysfunction. The pathophysiology of DR progression is still uncertain. This study investigates the function of beta-carotene (PBC) originating from palm oil mill effluent in the treatment of diabetes in mice. An intraperitoneal injection of streptozotocin (35 mg/kg) was used to induce diabetes, which was then accelerated by an intravitreal (i.vit.) injection of STZ (20 µL on day 7). PBC (50 and 100 mg/kg) and dexamethasone (DEX: 10 mg/kg) were also administered orally (p.o.) for 21 days. At various time intervals, the optomotor response (OMR) and visual-cue function test (VCFT) responses were evaluated. Biomarkers, such as reduced glutathione (GSH), thiobarbituric acid reactive substances (TBARSs), and catalase activity were determined in retinal tissue samples. DR significantly lowers the spatial frequency threshold (SFT) and time spent in the target quadrant (TSTQ), increases the reaching time in the visual-cue platform (RVCP), lowers retinal GSH and catalase activity levels, and elevates TBARS levels. The treatments of PBC and DEX also ameliorate STZ-induced DR alterations. The potential ameliorative activity of PBC in DR is attributed to its anti-diabetic, anti-oxidative, and control of blood–retinal barrier layer properties.

## 1. Introduction

Diabetic retinopathy (DR) is one of the common diabetic complications. DR mainly occurs due to micro-vascular damage, including the blood–retina barrier and retinal layer dysfunctions. DR is also developed by the macular degeneration of the retinal layer in the eyes [1]. The changes in retinal microvascular blood–retina barrier functions show visual impairments, along with hypertension, obesity, stroke, Parkinson’s disease, and dementia symptoms and vice versa [2,3]. Various toxins, such as ivermectin, enrofloxacin, ethylene glycol, baclofen hyaluronic acid, and 5-hydroxytryptophan, are documented to produce retinal damage that causes blindness [4,5]. Furthermore, chronic exposure to a retinal toxin, such as hydroxychloroquine, thioridazine, deferoxamine topiramate, and metronidazole, including streptozotocin (STZ), causes DR. STZ also causes central visual loss, reduced-color vision, central scotomata, macular degenerations, and optic neuropathy, including DR [6,7,8]. Through experimentation, it has been found that STZ is a causal factor in both diabetes and DR [9]. The intraperitoneal (i.p.) administration of STZ causes diabetes mellitus via the destruction of the beta-cell function of islets of Langerhans. However, the local administration of STZ readily interacts with various cellular components leading to the impairment of energy metabolism. These reactions were evidenced to cause Alzheimer’s disease, vascular dementia, and diabetic retinopathy [10,11]. Furthermore, STZ adducts, i.e., methyl-nitrosourea moiety, destroy DNA and activate the tissue plasminogen activator (tPA), leading to enhanced microvascular leakage and the cell death process [12,13]. These processes are known to increase the progression of DR. However, the exact molecular mechanism in the progression of DR and the alteration of these pathophysiological events remain unclear.

The clinical evidence reveals that the expression of free radical and pro-inflammatory genes, vascular endothelial growth factor (VEGF), and other proteins, such as cytokines, angiotensins, and endothelin peptides, cause the progression of DR via the regulation of blood–retina barrier functions [14]. The pre-clinical evidence also presents similar mechanisms in experimental animals, such as mice, via the alteration of neurovascular and microvascular functions [15,16]. The alteration of endothelial dysfunctions is the initial event in the progression of DR [17]. Beta-carotene (BC) is a precursor of pro-vitamin A [18,19]. It is evidenced to reverse vascular complications, such as atherosclerosis, via decreasing hepatic lipid secretion [19,20], and diabetic vascular dementia [18], including retinal vasculature [21]. Moreover, palm oil mill effluent-derived beta-carotene (PBC) also reduces vascular complications in obesity, cystic fibrosis, Alzheimer’s disease, vascular dementia, and diabetic neuropathic pain via metabolic regulatory functions [18,22,23,24,25]. PBC also attenuates non-insulin-dependent diabetes mellitus [26]. The richest sources of BC are carrots, spinach, lettuce, tomatoes, sweet potatoes, broccoli, and palm products [25,27]. Certain waste products also produce a high yield of beta-carotene and other vitamins [18,28,29]. Palm oil mill effluent (POME) is also one of the major, rich sources of beta-carotene [30]. Naturally, BC possesses free radical scavenging activity, prevents lipid peroxidative actions, and regulates gene expressions [31,32]. However, the role of PBC in DR is unknown. As a result, thus study is designed to investigate the involvement of PBC in STZ-induced DR in mice.

## 2. Results

### 2.1. Effect of PBC in the OMR Test as Blood–Retina Barrier Function-Associated Visual Acuity Actions

**Figure 1:**
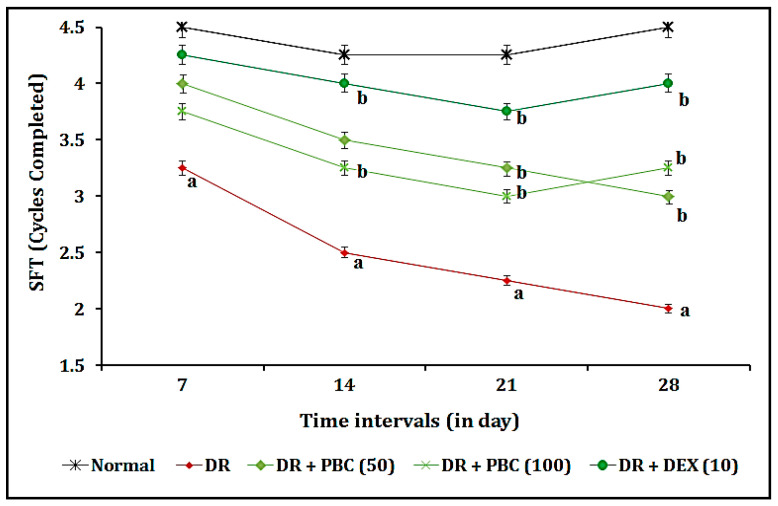
Effect of palm oil mill effluent-derived beta-carotene on streptozotocin-induced diabetic retinopathy-associated visual acuity functions in optomotor response test. The numbers in parentheses represent a dose of mg/kg. The results are presented as the mean SEM, with *n* = 10 mice per group. ^a^ *p* < 0.05 versus the control group; ^b^ *p* < 0.05 versus the DR group. Abbreviations: DEX stands for dexamethasone; DR stands for diabetic retinopathy; PBC stands for palm oil mill effluent-derived beta-carotene; and SFT stands for spatial frequency threshold.

The administration of STZ (20 µL; i.vit.) in diabetic mice expressed a statistically significant (*p* < 0.05) visual dysfunction as an indication of a decrease in SFT values, when compared to the normal group, due to changes in blood–retina barrier functions. The oral administration of PBC (50 and 100 mg/kg) for 21 consecutive days expressed the statistically significant attenuation of the OMR test response in a dose-dependent manner, when compared to the DR group. This effect resembled that of the DEX (10 mg/kg; p.o.) treatment group. The OMR test results indicate that the ameliorative effects on visual acuity actions are less pronounced in the DEX treatment groups as compared to PBC. The results of the data are illustrated in Figure 1.

### 2.2. Effect of PBC in VCFT on Blood–Retina Barrier Functions

**Figure 2:**
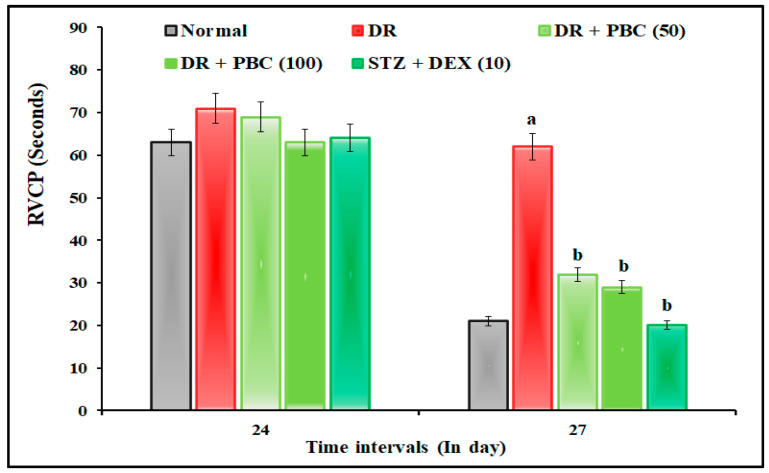
Effect of palm oil mill effluent-derived beta-carotene on streptozotocin-induced diabetic retinopathy -associated visual-cue functions test. The numbers in parentheses represent a dose of mg/kg. The results are presented as the mean SEM, with *n* = 10 mice per group. ^a^ *p* < 0.05 versus normal group; ^b^ *p* < 0.05 versus the DR group. Abbreviations: DEX stands for dexamethasone; DR stands for diabetic retinopathy; PBC stands for palm oil mill effluent-derived beta-carotene; RVCP stands for reach the visual-cue platform; and Sec stands for seconds.

**Figure 3:**
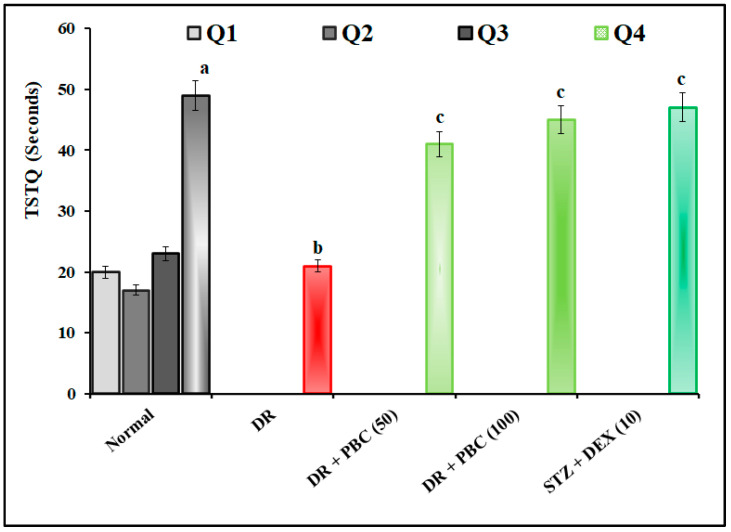
Effect of palm oil mill effluent-derived beta-carotene on streptozotocin-induced diabetic retinopathy-associated time spent in target quadrant in visual-cue function test. The numbers in parentheses represent a dose of mg/kg. The results are presented as the mean SEM, with *n* = 10 mice per group. ^a^ *p* < 0.05 versus the Q_1_ normal group; ^b^ *p* < 0.05 versus the Q_4_ normal group; ^c^ *p* < 0.05 versus the DR group. Abbreviations: DEX stands for dexamethasone; DR stands for diabetic retinopathy; PBC stands for palm oil mill effluent-derived beta-carotene; Q stands for quadrant; Sec stands for seconds; and TSTQ stands for time spent in the target quadrant.

The administration of STZ (20 µL; i.vit.) in diabetic mice expressed the statistically significant (*p* < 0.05) visual dysfunction as an indication of an increase in the time of RVCP and a decrease in TSTQ levels compared to normal mice. These changes were attributed to alterations in blood–retina barrier functions. When compared to the DR group, the oral treatment of PBC (50 and 100 mg/kg) for 21 successive days drastically reduced VCFT responses. This therapeutic impact was comparable to the DEX (10 mg/kg; p.o.) treatment group. PBC, with respect to DEX treatment groups, showed less ameliorative effects in blood–retina barrier functions in the VCFT test. The result of the data are illustrated in Figure 2 and Figure 3.

### 2.3. Effect of PBC on Biomarker Estimations

On the 28th day, the tail-vein method was used to collect blood samples from the mice. An Accu-Chek glucometer was used to measure the fasting blood glucose levels of the mice. Then, the animals were euthanized and retinal tissues were obtained to determine GSH, TBARS, and total protein levels. The following sections explain the outcomes of the biomarker alterations.

### 2.4. Effect of PBC on Blood Glucose Level Changes

The administration of STZ (20 µL; i.vit.) to diabetic mice expressed statistically significant (*p* < 0.05) diabetic mellitus as an indication of increased fasting blood glucose levels compared to a normal animal. When compared to the STZ group, the oral administration of PBC (50 and 100 mg/kg) for 21 successive days significantly attenuated the STZ-induced blood glucose levels in a dose-dependent manner. The results were found to be similar to the DEX (10 mg/kg; p.o.) treatment group. The changes in blood glucose levels were less pronounced in the PBC group compared to the DEX treatment groups. The results of the data are expressed in Table 1.

### 2.5. Effect of PBC on Retinal Tissue Biomarker Changes

The administration of STZ (20 µL; i.vit.) to diabetic mice expressed statistically significant (*p* < 0.05) retinal tissue biomarker changes as an indication of a decrease in GSH and catalase activity levels, as well as an increase in TBARS levels, when compared to a normal animal. When related to the STZ group, the oral administration of PBC (50 and 100 mg/kg) for 21 consecutive days significantly attenuated the DR associated with biomarker changes. This was comparable to the DEX (10 mg/kg; p.o.) treatment group. Compared to the DEX treatment groups, the PBC group presented reduced tissue biomarker changes. The results of the data are expressed in Table 2.

## 3. Discussion

The intravitreal administration of STZ (20 µL; i.vit.) in diabetic mice demonstrated a significant (*p* < 0.05) indication of DR via the changes in blood–retina barrier functions. The manifestations of DR were microaneurysm, intraretinal hemorrhage, exudate, macular edema, macular ischemia, and neovascularization [33,34]. Furthermore, STZ altered visual acuity by decreasing the SFT value in the OMR test, increasing the time of RVCP, and decreasing the TSTQ values in the VCFT test. Furthermore, it also reduced the GSH and catalase activity levels and increased the TBARS level in retinal tissue. These results are similar to other research reports [35,36]. In diabetic mice, the oral treatment of PBC (50 and 100 mg/kg) for 21 days substantially reduced DR associated with visual acuity and biomarker alterations. This effect was similar to that of the DEX (10 mg/kg) treatment group.

Our results indicate that DR conditions cause oxidative stress and membrane lipid peroxidation via the alteration of enzymatic and non-enzymatic defense systems of retinal tissues (blood–retina barrier changes) [37]. Chronic diabetes enhances cellular oxidative stress via the reduction in the reduced form of glutathione (γ-glutamyl-cysteinyl glycine) [38]. Furthermore, type-2 diabetes enhances the impairment of glucose metabolism along with reducing glutathione contents. It also coincides with a higher level of GSH deficiency and is reported in the pathogenesis of type-2 diabetes mellitus and its complications, such as DR [39,40]. Moreover, DR, with the complication of type-2 diabetes mellitus, causes blood–retina barrier damage associated with lipid peroxidation with a correlation to apoptosis [41]. The retinal tissues are highly susceptible to membrane lipid peroxidation and auto-oxidation due to the presence of a high quantity of polyunsaturated fatty acids. The present results indicate that PBC may have the ability to protect retinal tissue by exerting potential anti-oxidant and anti-lipid peroxidative effects. Furthermore, the pharmacokinetic profile of beta-carotene revealed that it is mainly converted and cleaved to form vitamin A in retinal tissue [42]. Vitamin A and carotenoids possess potent antioxidant properties and protect the cells against free radical actions [43]. Additionally, carotenoid groups also possess multidrug resistance (MDR)-reversing activity, which is responsible for drug efflux transporter’s actions [44]. Carotenoids are also known to reduce blood–retinal barrier breakdown and vascular leakages to prevent DR progression [45]. However, beta-carotene’s role in drug-transport actions via reversing MDR and drug efflux transporter’s actions remains unknown.

Furthermore, lipid peroxidation forms hydrogen peroxide and enhances the release of glutamate content in neurovascular tissue [46,47]. Catalase is a very common enzyme and is present in all types of cells, including retinal cells. Physiologically, endogenous catalase enzymes protect the retinal cells from free radicals associated with oxidative damage in the blood–retina barrier. Moreover, additional defense enzymes also play a role in retinal cell-protective functions, such as glutathione peroxidase and superoxide dismutase [48,49]. The administration of catalase therapy is also evidenced to protect the oxidative stress-associated progression of DR [50,51,52]. The reduced form of glutathione is present in various mammalian cells, including retinal tissue, and it contributes to the cellular defense system against oxidative free radicals [53]. Furthermore, it also maintains the integrity of the cell membrane due to the presence of an active thiol-disulfide bond [54]. Natural compounds’ action of enhancing endogenous enzymes reduced glutathione and regulated extracellular thiol–disulfide homeostasis in various stages of DR [40,55]. Similarly, TBARS, i.e., lipid peroxidation with oxygen free radicals, indicated oxidative stress. The elevation of lipid peroxidation products was observed in retinal tissues. The results of the present study also reveal that PBC ameliorates DR via anti-lipid peroxidative action in diabetic mice and are similar to the results in other reported works [56]. Anti-lipid peroxidative molecules, such as tocotrienol-rich fractions, are also known to attenuate DR in rats [57].

The natural agent PBC is shown to reduce oxidative stress in neurons and blood vessels, including retinal tissue [18,58,59]. Moreover, the administration of BC is also known to enhance catalase activity to enhance cytoprotective actions [60]. Moreover, DEX also possesses potential pleiotropic actions against various diseases, including DR, due to its anti-inflammatory, anti-leukemic, and immunosuppressive actions [61,62]. In diabetic rats, DEX, a type of corticosteroid, can block the intracellular inflammatory signaling of lipid mediators, such as prostaglandins and leukotrienes [63]. Moreover, it inhibits leukocyte accumulation and vascular reactivity in the retina via the inhibition of the vascular endothelial growth factor and intercellular adhesion molecule-1 expression [64]. Various preclinical models have reported the pleiotropic activity of DEX, which suggests that corticosteroids could be used for the treatment of DR in humans [65]. However, the major drawback of DEX usage causes depression via the modulation of cellular glucose metabolism and AMPK/mTOR signaling pathways [66]; alterations in the vascularization process via the modification of progesterone receptor actions [67]; and enhances muscular atrophy [68]. Hence, the therapeutic usage of DEX remains challenging due to its lack of safety and multiple adverse effects.

Experimentally, synthetic glucocorticoid, i.e., DEX, is used for the treatment of DR due to its anti-inflammatory and immunological defensive properties [69,70]. PBC also possesses multi-targeted actions, such as DEX, and it protects from cellular oxidative damage in multiple diabetic complications [71,72]. Furthermore, the pleiotropic action potentials of beta-carotene reduce the risk factor of cardiovascular disease, heart disease, stroke, cancer, associated cell death, and mortality [72]. In addition, beta-carotene also regulates the 15,15′-mono-oxygenase gene and puromycin-induced nephrosis and retinol damage in rats [73]. Beta-carotene is also reported to attenuate the vascular complication of non-insulin-dependent diabetes mellitus patients via the reduction in reactive oxygen species with the increase in plasma and erythrocyte glutathione and erythrocyte glutathione peroxidase levels [26]. Furthermore, our recent study report also revealed that beta-carotene attenuates the neurovascular complications associated with vascular dementia [18] and diabetic neuropathic pain via the inhibition of matrix metalloprotease-13 action [25]. Comparatively, DEX has a major role in the amelioration of DR, whereas PBC also significantly contributes to the amelioration of retinopathy in diabetic mice models. However, the pure form of BC may contribute a better efficacy to attenuate DR based on its potential multi-targeted actions. Hence, PBC can be used for DR after a more extensive study on larger-animal vertebrate models with different pathological conditions.

## 4. Materials and Methods

### 4.1. Chemicals and Reagents

POME samples were obtained during the high-crop season from Palm Oil Mill Sdn. Bhd. in Penang (latitude: 5.285153 and longitude: 100.456238), Malaysia. Furthermore, samples were stored in an airtight, opaque container and kept in a chiller at 277 K (3.85 °C). The concentrated oil was obtained from POME by the solvent extraction method, as described by Ahmad et al. [74]. Briefly, organic solvent, i.e., n-hexane, was used for obtaining the concentrated oil with the close monitoring of the solvent ratio, mixing speed and time, and pH. PBC was separated using three different adsorbents, i.e., silica gel, florisil, and alumina. In a nutshell, 10 g of adsorbents were placed into the column (10 mm internal diameter and 13.5 mm length). The n-hexane was used to equilibrate the column. The yield of POME was approximately 2.5 g of concentrated oil (oil:adsorbent ratio 10:3 *w*/*w*). Then, it was loaded in the column chromatography to collect the PBC with the sequential addition of the mobile phase, i.e., 100 mL of n-hexane (non-polar solvent) and 100 mL of ethanol (polar solvent) with variable ratios of extracted oil:adsorbent (*w*/*w*), i.e., 1:4, 1:5, and 1:6 [28]. The fractions (eluted) of carotene content were determined as the described method for the PORIM test [75]. In brief, 0.1 g of the samples were dissolved in 25 mL of n-hexane and measured at 446 nm at 30, 40, and 50 °C using a spectrophotometer. The isolation of PBC and characterization with HPLC techniques were described in another article (unpublished data). About 67 mg of PBC was quantified in 0.1 g of samples, and it was used in this study. Dexamethasone sodium phosphate injection (Decadron Phosphate^®^, Mumbai, India) was procured from a pharmacy. Reduced glutathione (GSH), 5,5′-dithiobis (2-nitrobenzoic acid) (Ellman’s reagent), 1,1,3,3-tetra methoxy propane (TMP), cobalt (II) chloride, thiobarbituric acid (TBA), Graham salt (i.e., sodium hexametaphosphate), hydrogen peroxide solution, Lowry’s reagent, Folin–Ciocalteu reagent, and bovine serum albumin were procured from Merck Sdn Bhd., Selangor, Malaysia. All the reagents were procured with analytical grades.

### 4.2. Animals Used

In this investigation, male Swiss mice (12 months old; 20–30 g) were used. Animals fed with a laboratory animal diet and mice were allowed to have free access to drinking water ad libitum. In the central animal house, a 12 h day and night cycle was maintained. The institutional animal ethics committee (IAEC approval no.: AUAEC/FOP/2022/08) authorized the experimental study design. The mice were cared according to AIMST University’s IAEC guidelines. The diabetic mice were produced with an intraperitoneal administration of STZ (35 mg/kg; day 1), as explained by Yuan et al. [76]. On the seventh day, the intravitreal injection of STZ (20 µL of 7% STZ) promoted the course of DR.

### 4.3. Estimation of Blood Glucose Level

The level of fasting blood sugar was determined using an Accu-Chek glucometer device, as described by Villena Gonzales et al. [77]. Briefly, blood samples were obtained using the tail-prick method. The blood glucose levels were recorded on days 0, 3, 7, and 28. The estimation of the blood glucose levels on the 28th day was performed following the assessment of their behavioral parameters. The mice were considered diabetic when the stable fasting blood glucose level exceeded 150 mg/dL or 8.3 mmol/L. A mortality rate, i.e., 10 to 12 percent, was observed for the induction of diabetic mice with stable elevated blood glucose levels. These diabetic mice were used for the testing of DR as per the requirement of the experimental design.

### 4.4. Experimental Design

Our investigation used five groups of male adult mice (*n* = 10/group). Group 1 was the naïve control group. Group 2 animals acted as the DR group. The test compounds’ PBC (50 and 100 mg/kg; p.o.) were treated in groups 3 and 4, respectively. PBC was administered for 21 consecutive days from the 8th day, respectively. Group 5 animals served as the dexamethasone (DEX, 10 mg/kg; p.o.) control group. DEX was administered for 21 days from the 8th day. PBC suspension was produced with 0.5% *w*/*v* carboxymethyl cellulose and administered orally by the oral gavage method. Glucose variability was observed in diabetic animals until the first 7 days. The progression of DR was accelerated by an intravitreal injection of STZ (20 µL of 7% STZ) on the 7th day. Then, PBC and DEX were administered from the 8th day of the study design. The visual acuity responses, i.e., optomotor response (OMR) and visual-cue function test (VCFT), were assessed with optokinetic (a fabricated device located in the Pharmaceutical Technology Laboratory, AIMST University) and Morris water-maze (a fabricated device located in Central Animal House, AIMST University) devices, respectively. The assessment of the OMR was recorded on days 7, 14, 21, and 28. The assessment of VCFT was performed from days 24 to 28.

### 4.5. Assessment of OMR Using an Optokinetic Device

**Figure 4:**
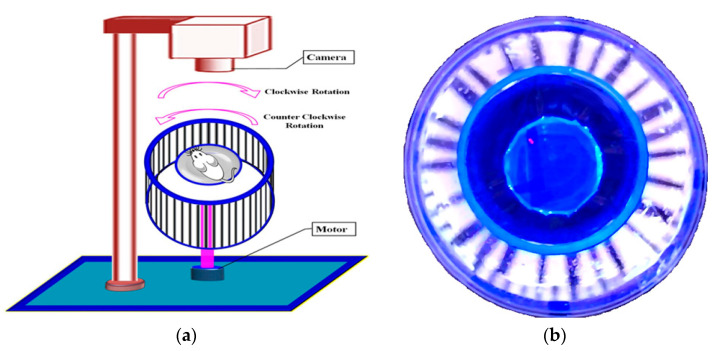
Illustration of optokinetic motor device for the assessment of mice visual acuity functions. Panel (**a**) presents the diagrammatic view of the optokinetic motor device. The outer rotating drum can revolve clockwise and anti-clockwise with a constant speed of 10 rpm. The recording device was placed on the top side of the optokinetic motor device. Panel (**b**) presents the top view of the optokinetic motor device, and the wall side has multiple light and dark strips to contrast the light against the mice’s vision, and helps to measure the visual acuity function.

The optokinetic motor response (OMR) was used for the assessment of DR-associated visual impairments. The OMR was conducted according to the method described by Prusky et al. [78], with the slight modification of Kretschmer et al. [79]. Briefly, the OMR device consisted of two concentric-circular champers. It was covered with a non-transparent square box (creating an inner-side dark environment). A white light-emitting diode (LED) strip light was placed on the inner wall of the square box. The inner wall of the large, circular chambers was composed of with multiple black and white stripes and positioned vertically. This was connected to a 10 revolutions per minute (rpm) motor to create a rotating drum. The non-revolving, smaller, transparent, circular chamber was placed at the center of the OMR device. The mice were placed in this smaller chamber. In this condition, mice can observe the outer black and white stripes’ movements. The visual acuity test was assessed based on the responses of the animals’ movements against the clockwise and anti-clockwise movements of the outer (transparent circular) chamber. The recorded animal-movement observation was spatial frequency threshold (SFT), i.e., known as a whole animal circular movement against the grid movement within the 4 min observation period. The assessments were repeated six times to achieve reproducible responses. The optokinetic motor device for the assessment of mice visual acuity functions has been illustrated in Figure 4.

### 4.6. Assessment of Visual-Cue Function Test (VCFT) by MORRIS Water-Maze Device

**Figure 5:**
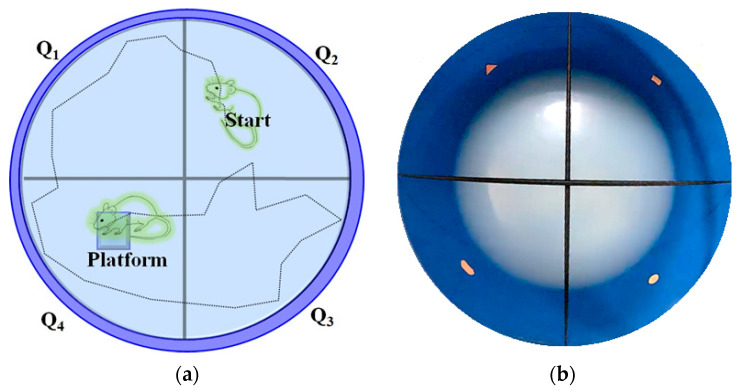
Illustration of Morris water-maze device for visual-cue function test (VCFT) response. Panel (**a**) presents the diagrammatic view of the Morris water-maze device expressing the quadrant (Q) arrangements. Animal-placement location is considered as a starting point. The location of the target quadrant (i.e., Q4) platform is considered the destination point for the mice. Panel (**b**) presents the top view of the Morris water-maze device and the wall of the Morris water-maze device has a multidimensional visual cue for the identification of each quadrant.

The visual-cue function test (VCFT) was assessed, as described by the method of Morris (1981) [80], with the modifications of Prusky et al. [81], and Sherwin and Glen [82] using the Morris water-maze water pool device. In summary, the Morris water maze was built as a circular water pool (120 cm in diameter and 45 cm in height) with a featureless inner surface. The inner wall of the water-maze test apparatus is composed of four external visual cues and is filled with water and mixed with non-toxic white-colored dye. The water temperature was kept constant at 23–25 °C. A white platform (10 × 10 cm square and 28 cm in height) was placed in one of the quadrants with an equal area, and the water level was maintained at 2 cm below the platform surface. At the center of the platform, a green flag was placed to raise visibility. In each experiment, the time it took to find the platform was recorded. The mice were employed for four days of training in each quadrant with the visual-cue platform. When the mice identified the platform, the animal was allowed to stay on it for an additional 10 s. If the animal did not find the visual-cue platform (reach the visual-cue platform named RVCP) within 120 s, they were directed to the platform and were allowed to stay on it for an extra 10 s. The visual-cue platform was removed from the water-maze tank on the final day, and a probing test was performed. The mice were engaged for 90 s to identify the location of the target platform. This time spent in the target guardant (TSTQ) was considered as the mice’s visual activity responses [81,82]. The Morris water-maze device for visual-cue function test (VCFT) response has been illustrated in Figure 5.

### 4.7. Estimations of Diabetic Retinal Tissue (Blood–Retina Barrier) Marker Changes

On the 28th day, following the collection of the behavioral data, the mice were sacrificed by the cervical dislocation procedure under an anesthetic condition. The eyeballs were extracted from the mice then the retina was immediately segmented. Briefly, the eyeballs were sectioned into two portions, i.e., anterior and posterior, with a scalpel. The half-circled inner-layer posterior portion of the eyeball containing the lens and epithelium pigments was isolated with the help of curved Dumont forceps by applying gentle pressure. Furthermore, non-retinal tissue and sclera were freed by using forceps. The isolated retinal tissue portions were immediately placed in ice-cooled phosphate buffer (pH–7.4). On the 29th day, the biomarkers, i.e., TBARS, GSH, and catalase activity levels, were determined in retinal tissue samples.

#### 4.7.1. GSH Estimation as an Indicator of Non-Enzymatic Oxidative Stress

The GSH levels in the retinal tissue were quantified, as described by Ellman [83]. In brief, a supernatant of the retinal tissue was produced and combined with 10% *w*/*v* tri-chloroacetic acid (1:1 ratio) to create protein precipitation. The mixtures were centrifuged at 4 °C for 10 min at 1000 revolutions per minute (rpm). A total of 2 mL of 0.3 M disodium hydrogen phosphate was added to 0.5 mL of clear aliquot. Then, 0.25 mL of freshly produced 0.001 M DTNB solutions were also added. Then, DTNB was dissolved in a solution of 1% *w*/*v* sodium citrate. The yellow-colored chromogen variability was measured as absorbance changes with a spectrophotometer at 412 nanometers (DU 640B Spectrophotometer, Beckman Coulter Inc., Brea, CA, USA). A standard curve was created based on the changes in the absorbance value with standard GSH (10–100 micromoles of GSH per mL). The overall result was reported in µmol of reduced glutathione (GSH) in every mg of tissue protein sample.

#### 4.7.2. TBARS Estimation as an Indicator of Lipid Peroxidation

The TBARS levels in retinal tissue were quantified, as described by Ohkawa et al. [84]. Briefly, 0.2 mL of homogenate supernatant was combined with 0.2 mL of 8.1% sodium, 0.2 mL of 8.1% sodium dodecyl sulfate, 1.5 mL of 30% acetic acid, and 1.5 mL of 0.8% TBA. The total volume wreached 4 mL with distilled water. Then, test tubes were incubated for 1 h at 90 °C. Subsequently, 1 mL of distilled water was added and centrifuged at 4000 rpm for 10 min. The pink-colored chromogen variations were measured as absorbance changes with a spectrophotometer at 532 nanometers (DU 640B Spectro-photometer, Beckman Coulter Inc., Brea, CA, USA). The overall result was reported in nmol of malondialdehyde (MDA) in every mg of tissue protein sample.

#### 4.7.3. Catalase Estimation as an Indicator of Enzymatic Oxidative Stress

The mice’s retinal layer tissue catalase enzyme activities were quantified, as described by Hadwan [85]. Briefly, 0.5 mL of aliquot was mixed with 0.5 mL of distilled water, and 1 mL of 10 mM hydrogen peroxide solution (pH 7.0, 50 mM) was added. The test tubes were vortexed further and then incubated at room temperature, i.e., 37 °C for 2 min. Then, 0.6 mL of working reagent was added, i.e., a mixture of cobalt, Graham salt, and sodium bicarbonate solutions in a 1:1:1.8 ratio. The mixture of the solution was immediately vortexed for 5 s and thereafter incubated at 37 °C for 10 min under dark conditions. The olive-green-colored chromogen [carbonato-cobaltate (III) complex] changes were recorded as absorbance variations using a spectrophotometer (DU 640B Spectrophotometer, Beckman Coulter Inc., Brea, CA, USA) at 440 nanometers. The total catalase enzyme activity was calculated using the formula:

Catalase activity (U/mL) = (2.303)/(t) log (δ O.D. standard)/(δ O.D. test)

Here, optical density (O.D.) denotes the change in absorbance per minute and ‘t’ represents the time in this formula. The catalase activity level was then calculated using an integrated calculation using mg of tissue protein. The catalase action was represented as a unit of catalase activity in every mg of tissue proteins.

#### 4.7.4. Estimation of Retinal Tissue Proteins

According to Lowry et al. [63], the total protein content in the retinal tissue was determined [86]. In brief, 0.15 mL of tissue supernatant was adjusted up to 1 mL with phosphate buffer before being combined with 5 mL of Lowry’s reagents in test tubes. The test tubes were incubated for 15 min at room temperature before adding 0.5 mL of Folin–Ciocalteu reagent, rapidly vortexed, and incubated for 30 min at room temperature. A spectrophotometer (DU 640B Spectrophotometer, Beckman Coulter Inc., Brea, CA, USA) at 750 nanometers was used to examine the purple-shade chromogen alterations. The standard curve contained 0.2–2.4 mg of bovine serum albumin per milliliter. The overall result was reported in mg of tissue protein in every gram of tissue sample.

### 4.8. Statistical Analysis

All results were presented as the mean standard deviations of the mean (SEM). The OMR and VCFT data were statistically evaluated using a two-way ANOVA test accompanied by the Bonferroni post hoc test. Furthermore, using Graph pad Prism version 5.0 software Dotmatics (R&D scientific software company, San Diego, CA, USA), the data for blood glucose, GSH, TBARS, and catalase activity levels were examined using one-way ANOVA followed by Tukey’s multiple range tests. A statistically significant outcome was defined as a probability (*p*) value of less than 0.05.

## 5. Conclusions

The results of the present study reveal that the treatment of PBC ameliorates DR-associated visual acuity responses in the OMR test and VCFT, along with the modification of STZ-induced biomarker changes, i.e., the enhancement of the GSH and catalase activity levels, as well as the reduction in the TBARS levels in retinal tissue. Hence, PBC can be used for the treatment of microvascular complications due to its potential free radical scavenging activity, anti-lipid peroxidative behavior, and activation of endogenous free radical defense enzymatic actions. However, this study will be extended to explore chemokine CXCL1-mediated neutrophil recruitment pathways with suitable regulators and experimental tools for the management of DR progression.

## Figures and Tables

**Table 1:** Effect of palm oil mill effluent-derived beta-carotene on streptozotocin-associated changes in blood glucose levels.

Groups	Day 0 (mmol/L)	Day 3 (mmol/L)	Day 8 (mmol/L)	Day 28 (mmol/L)
Normal	6.1 ± 0.5	6.4 ± 0.6	6.3 ± 0.6	6.5 ± 0.5
DR	6.2 ± 0.7	24.7 ± 1.4 ^a^	25.3 ± 1.3 ^a^	29.7 ± 1.1 ^a^
DR + PBC (50)	6.3 ± 0.5	25.8 ± 1.1 ^a^	26.3 ± 1.0 ^a^	11.2 ± 1.4 ^b^
DR + PBC (100)	6.4 ± 0.4	24.2 ± 1.2 ^a^	24.2 ± 1.5 ^a^	9.4 ± 1.2 ^b^
DR + DEX (10)	5.9 ± 0.3	23.1 ± 1.8 ^a^	25.4 ± 1.4 ^a^	8.2 ± 1.3 ^b^

The numbers in parentheses represent a dose of mg/kg. The results are presented as the mean SEM, with *n* = 10 mice per group. ^a^ *p* < 0.05 versus the normal group; ^b^ *p* < 0.05 versus the STZ group. Abbreviations: STZ stands for streptozotocin; DEX stands for dexamethasone; and PBC stands for palm oil mill effluent-derived beta-carotene.

**Table 2:** Effect of palm oil mill effluent-derived beta-carotene on retinal tissue biomarker changes.

Groups	GSH (µmol/mg of Protein)	TBARS (nmol/mg of Protein)	Catalase (U/mg of Protein)
Normal	24.8 ± 1.5	1.3 ± 0.05	15.2 ± 1.4
DR	7.94 ± 1.4 ^a^	3.4 ± 0.08 ^a^	2.9 ± 1.3 ^a^
DR + PBC (50)	13.3 ± 1.2 ^b^	2.3 ± 0.03 ^b^	5.2 ± 0.8 ^b^
DR + PBC (100)	10.5 ± 1.4 ^b^	1.9 ± 0.04 ^b^	4.8 ± 0.3 ^b^
DR + DEX (10)	8.6 ± 1.7 ^b^	1.6 ± 0.07 ^b^	4.4 ± 1.2 ^b^

The numbers in parentheses represent a dose of mg/kg. The results are presented as the mean SEM, with *n* = 10 mice per group. ^a^ *p* < 0.5 versus the normal group. ^b^ *p* < 0.5 versus the DR group. *Abbreviations:* DEX stands for dexamethasone; GSH stands for reduced glutathione; PBC stands for palm oil mill effluent-derived beta-carotene; STZ stands for streptozotocin; and TBARS stands for thiobarbituric acid reactive substances.

## Data Availability

The data presented in this study are available on request from the corresponding author.
